# Mechanics of knee meniscus results from precise balance between material microstructure and synovial fluid viscosity

**DOI:** 10.1371/journal.pone.0304440

**Published:** 2025-09-11

**Authors:** Camilo A. S. Afanador, Stéphane Urcun, Ivo F. Sbalzarini, Stéphane P. A. Bordas, Olga Barrera, Mohammad Mahdi Rajabi, Romain Seil, Anas Obeidat

**Affiliations:** 1 Department of Engineering, Institute of Computational Engineering, University of Luxembourg, Esch sur Alzette, Luxembourg; 2 Aix-Marseille University, LMA-CNRS, Centrale Marseille, France; 3 Clyde Visiting Fellow, Department of Mechanical Engineering, The University of Utah, Salt Lake City, Utah, United States of America; 4 Dresden University of Technology, Faculty of Computer Science, Dresden, Germany; 5 Max Planck Institute of Molecular Cell Biology and Genetics, Dresden, Germany; 6 Center for Systems Biology Dresden, Dresden, Germany; 7 Cluster of Excellence Physics of Life, TU Dresden, Dresden, Germany; 8 School of Engineering Computing and Mathematics, Oxford Brookes University, Oxford, United Kingdom; 9 University and Department of Engineering Science, University of Oxford, Oxford, United Kingdom; 10 Department of Orthopaedic Surgery, Centre Hospitalier de Luxembourg - Clinique d’Eich, Luxembourg, Luxembourg; 11 Luxembourg Institute of Research in Orthopaedics, Sports Medicine and Science (LIROMS), Luxembourg, Luxembourg; 12 Human Motion, Orthopaedics, Sports Medicine and Digital Methods, Luxembourg Institute of Health, Luxembourg, Luxembourg; University of Zaragoza, SPAIN

## Abstract

The meniscus plays a crucial role in the biomechanics of the knee, serving as load transmitter and reducing friction between joints. Understanding the biomechanics of the meniscus is essential to effective treatment of knee injuries and degenerative conditions. This study aims to elucidate the relationship between the porous microstructure of the human knee meniscus and its biomechanical function, specifically focusing on fluid dynamics at the pore scale. Here, we use two central-meniscus samples extracted from a human knee and reconstruct high-resolution geometry models from μ-CT scans. By eroding the channels of the original meniscus geometry, we simulate perturbed microstructures with varying porosities ( **53%** to **80%**), whilst preserving the connectivity of the porous structure. We numerically solve for the fluid dynamics in the meniscus using a mesh-free particle method, considering various inlet pressure conditions, characterising the fluid flow within the microstructures. The results of the original microstructure associated with a physiological dynamic viscosity of synovial fluid are in accordance with biophysical experiments on menisci. Furthermore, the eroded microstructure with a **33%** increase in porosity exhibited a remarkable **120%** increase in flow velocity. This emphasises the sensitivity of meniscus physiology to the porous microstructure, showing that detailed computational models can explore physiological and pathological conditions, advancing further knee biomechanics research.

## Introduction

The biomechanical functions of menisci in the knee include load transmission, friction reduction between joints [1–3], and possibly shock absorption. The shock-absorbing function of the meniscus, while commonly cited, is however a subject of ongoing debate [4]. Prior to 1980, menisci were often considered superfluous and were frequently removed after injury, leading to negative consequences such as arthrosis in patients [3]. The understanding of meniscus function has improved significantly, with multiple aspects believed to contribute to their energy dissipation ability, including the geometry and the biochemical composition of the microstructure [1–3,5–10].

In this study, menisci are considered as a porous medium. This porous medium contains a structural solid scaffold mainly composed of type I collagen (75%), large hydrophilic molecules termed proteoglycans (6%) [5]. This solid scaffold is saturated by synovial fluid, the dynamic viscosity of which depends on the meniscus’ health and its physiological conditions [11].

The main component of the structural scaffold, the collagen fibers which ensure its mechanical integrity [12], contributes to energy dissipation by its structure ensuring a circumferential tensile stress [6,7]. The chemical composition of the structural scaffold, particularly the presence of proteoglycans, plays a crucial role in defining the shock-absorption properties [5]. Proteoglycan are large hydrophilic molecules that ensure tissue elasticity under small loads [5]. Under larger loads (>100kPa), the interstitial fluid starts to flow, causing menisci to behave as a viscous porous medium [8].

We only found few quantitative studies that used numerical simulations to relate the mechanical and structural properties of the meniscus [13–15]. These studies have provided insights into the viscoelastic properties and the anisotropy of permeability due to collagen fiber orientation in the meniscus [15,16].

Advances in computing power and microscale characterization techniques, such as micro-computed tomography (*μ*-CT), magnetic resonance velocimetry (MRV), and X-ray microtomography (XMT), have enabled the study of fluid flow and transport processes at the microscopic scale in porous media [17–20]. These techniques allow accurate reconstruction of the pore structure of porous media in three dimensions, which can then be used as input for computational fluid dynamics (CFD) simulations at the pore scale [21,22]. This study specifically aims to understand how microstructure and fluid viscosity affect meniscus biomechanics. Here, we use these computational advances to disentangle the contributions of chemical, structural, and geometric factors to the energy dissipation capability of human menisci, building on existing literature and research [1–3,5–10].

Here we use two central-meniscus samples extracted from a human knee imaged by high-resolution *μ*-CT (6.25μm resolution). For these samples, we reconstructed a 3D computational geometry model using an implicit immersed boundary technique. We then solve an alternative formulation of the incompressible Navier-Stokes equations (the Entropically Damped Artificial Compressibility equations) inside the resulting microstructures, using different inlet pressure boundary conditions representative of different loading scenarios.

We find that by surpassing a certain pressure threshold, the flow in the meniscus qualitatively changes from a slow creeping flow to a pressure-dominated laminar flow. The existence of these two qualitatively different flow regimes is typically associated with healthy menisci. Increasing the porosity of the meniscus microstructure enhances hydration, but excessive porosity leads to pathological responses, interfering with joint function, affecting tissue nutrient exchange, and leading to joint degeneration [23–27]. In the context of osteoarthritis, which leads to a degenerated meniscus, a decrease in collagen content alongside an increase in water content were observed both in histology and imaging [28]. Translated to a poromechanical framework, as modelled here, this leads to a thinning of the solid scaffold and therefore an increase of the porosity. Additionally, osteoarthritis provokes a decrease in synovial fluid viscosity [29]. We elucidate the mechanism behind this by eroding the meniscus geometry to construct microstructures ranging from a porosity of 53% to a porosity of 80% at constant connectivity of the pore structure. Of course, an artificially increased porosity as high as 80% is not physiological. However, the purpose of dilating the microstructure to such limits is to extrapolate the impact of the meniscus’ microstructural characteristics on its macroscopic properties. We find that eroded geometries: (i) no longer exhibit the two flow regimes and (ii) display a non-linear relationship between porosity and velocity magnitude. For instance, a 30% increase in porosity leads to a 120% increase in velocity magnitude.

Taken together, the fully resolved pore-scale computer models of meniscus geometry and direct numerical fluid flow simulations within them allow us to accurately capture the geometry–mechanics trade-off in menisci. On the one hand, fluid flow in a meniscus should be sufficiently slow and dampened for it to exhibit shock absorbing properties. On the other hand, though, some flow must exist in order to transport metabolites and nutrients in and out of the tissue. This requires a fine-tuned balance between the geometric microstructure of the meniscus and the viscosity of the synovial fluid. Using the present simulations, we were able to understand this balance mechanistically and predict impact of meniscus microstructure on mechanical function under fully controlled conditions.

## Materials and methods

### Entropically Damped Artificial Compressibility (EDAC)

In the realm of simulating the incompressible Navier-Stokes equations, Clausen [30] introduced the EDAC method. This method paved the way for the explicit simulation of these equations. Within the EDAC formulation, a new equation governing the evolution of pressure *p* is introduced. This equation is derived from the thermodynamics of the system while maintaining a fixed density *ρ*. Remarkably, the EDAC method exhibits convergence to the incompressible Navier-Stokes equations when operating at low Mach numbers, i.e., at flow velocities much smaller than the speed of sound, and it maintains consistency both at low and high Reynolds numbers. Consequently, it becomes feasible to explicitly solve both the momentum equation and the pressure evolution equation [31], which are given in the Eulerian frame of reference as:

$$\rho \frac{du_{i}}{dt}+u_{j}\frac{\partial u_{i}}{\partial x_{j}}=-\frac{\partial p}{\partial x_{i}}+\frac{\partial \tau_{ij}}{\partial x_{j}}-\frac{\chi }{\eta }(u_{i}-u_{(oq)i})$$ (1)

$$\frac{dp}{dt}+u_{i}\frac{\partial p}{\partial x_{i}}=-{c_{s}}^{2}\rho_{o}\frac{\partial u_{i}}{\partial x_{i}}+\nu \frac{\partial^{2}p}{\partial x_{i}x_{i}}$$ (2)

$$\tau_{ij}=\mu \left(\frac{\partial u_{i}}{\partial x_{j}}+\frac{\partial u_{j}}{\partial x_{i}}-\frac{2}{3}\delta_{ij}\frac{\partial u_{k}}{\partial x_{k}}\right).$$ (3)

This approach involves the implicit penalisation of the computational domain, achieved by the indicator function χ that identifies the areas occupied by the solid geometry denoted as *O*:

$$\chi (x)=\left\{ \begin{array}{cc} 1 & \text{if}\;\;\;x\in O, \end{array}\right.$$ (4)

For conciseness of notation, the equations above are written in Einstein summation notation. Eq (1) is the momentum equation. In this equation:

- *ρ* is the fluid density, representing the mass per unit volume.
- $\frac{du_{i}}{dt}$ is the time derivative of the velocity component *u*_*i*_, indicating how the velocity changes over time.
- $u_{j}\frac{\partial u_{i}}{\partial x_{j}}$ represents advection, describing how the velocity varies spatially within the fluid.
- $\frac{\partial p}{\partial x_{i}}$ is the pressure gradient, the driving force for fluid flow.
- $\frac{\partial \tau_{ij}}{\partial x_{j}}$ represents the stress divergence, accounting for viscous forces within the fluid.
- *u*_(*oq*)*i*_ is the velocity of the solid body, i.e., the walls of the meniscus.
- η=αϕ is the normalized viscous permeability, where *ϕ* is the porosity of the medium and *η* is a small parameter, with 0<ϕ≪1 and 0<η≪1 .
- *μ* is the dynamic viscosity of the fluid.
- χ is the penalization mask function, which enforces boundary conditions and represents solid regions as defined in Eq (4).

Eq (2) is the pressure evolution equation. There:

- $\frac{dp}{dt}$ is the time derivative of pressure, indicating the rate of pressure change over time.
- $u_{i}\frac{\partial p}{\partial x_{i}}$ is the advection term for pressure, showing how pressure is transported by the fluid flow.
$-c_{s}^{2}\rho_{o}\frac{\partial u_{i}}{\partial x_{i}}$ is the acoustic term, related to the compressibility of the fluid, where *c*_*s*_ is the speed of sound and $\rho_{o}$ is the reference density.
- $\nu \frac{\partial^{2}p}{\partial x_{i}x_{i}}$ represents diffusion, allowing pressure to diffuse through the fluid.

Finally, Eq (3) defines the shear stress tensor:

- *μ* is the dynamic viscosity.
- $\frac{\partial u_{i}}{\partial x_{j}}+\frac{\partial u_{j}}{\partial x_{i}}$ represents the rate of strain tensor, which describes the deformation of the fluid.
- $\frac{2}{3}\delta_{ij}\frac{\partial u_{k}}{\partial x_{k}}$ is the volume change correction, subtracting the isotropic part of the deformation to maintain consistency with the fluid’s compressibility.

The EDAC method converges to the incompressible Navier Stokes equations at low Mach numbers, and the equations can be solved explicitly in a Lagrangian or Eulerian frame of reference, please refer to [30].

### Discretisation-Corrected Particle Strength Exchange (DC-PSE)

To simulate fluid flow through the meniscus, we use the mesh-free Discretisation-Corrected Particle Strength Exchange (DC-PSE) method [32]. DC-PSE is a numerical method for consistently discretising differential operators on Eulerian or Lagrangian particles, which represent the mathematical collocation points on which the continuous fields are sampled. DC-PSE is based on the approximation of a sufficiently smooth function $f_{\epsilon }(\vec{x})$ with a kernel η(),

$$f(\vec{x})\approx f_{\epsilon }(\vec{x})=\int_{\Omega }f(\vec{y})\eta_{\epsilon }(\vec{x}-\vec{y})\;d\vec{y},$$ (5)

where *ε* is the radius of the kernel, which is defined as:

$$\eta (\vec{x})=\left\{ \begin{array}{cc} \sum_{i,j}^{i+j<r+m+n}a_{i,j}x^{i}y^{j}e^{-x^{2}-y^{2}} & \sqrt{x^{2}+y^{2}}<r_{c}\\ 0 & \text{ otherwise, } \end{array}\right.$$ (6)

where the polynomial coefficients *a*_*i*,*j*_ are determined from discrete moment conditions evaluated at run-time on the given set of particles [32]. The discrete moment conditions are derived using Taylor series expansion such that the operators are consistent to a desired order of convergence. We encourage the reader to refer to Ref. [31] for further discussion regarding the EDAC formulation of the DC-PSE method, where the method is validated with different benchmarks and a convergence and accuracy studies are provided.

### Simulation setup, initial condition and boundaries

The DC-PSE simulations are performed in the Eulerian frame of reference with low Reynolds number Re≪1. The simulations use DC-PSE operators of convergence order 3, a particle interaction cutoff radius of 3.1ϵ, and second-order Adams Bashforth-Moulton adaptive time integration.

The boundary conditions are chosen according to the literature. At the inflow, we apply inlet pressure boundary conditions in *z* directions with a range of values (1.1 bar to 2.5 bar) based on previous studies that examined synovial fluid pressure under various joint loading scenarios [6,8,11]. This range allows us to validate two distinct flow regimes: a visco-elastic creeping flow observed below the critical pressure of approximately 1.7 bar, and a laminar porous-permeable flow above this threshold. Periodic boundary conditions are applied in the *x* and *y* directions, while outflow conditions are implemented in the *z* direction. In the literature, various estimates for the fluid-dynamic viscosity *μ* have been provided [33,34]. Bera et al. [34] found that there is no established standard for the dynamic viscosity of the synovial fluid, with a range of values from $0.7\;\cdot \;10^{-3}$ to $3.5\;\cdot \;10^{-3}$ Pa·s. Meanwhile, Fu et al. [33] determined that the viscosity of the synovial fluid in the meniscus with periprosthetic joint infection can be as high as $1.5\;\cdot \;10^{-2}\;\text{Pa}\;\cdot \;\text{s}$. Galandakova et al. [11] conducted a study to determine the synovial fluid viscosity in the knee joint using a Vibro viscometer and concluded the median viscosity value for a sample of 22 healthy menisci is $7.3\;\cdot \;10^{-3}\;\text{Pa}\;\cdot \;\text{s}$. We use this viscosity value as a reference in our study.

The spatial domain is discretised using particles, with (128×128×256) particles in the *x*, *y*, and *z* directions, respectively. The DC-PSE solver for EDAC formulation is integrated into the C++ open-source high-performance computing platform OpenFPM. [35], (see Mathematical modelling). The C++ code is compiled using gcc 8.3.0 and OpenMPI 3.1.3 on 64-core AMD EPYC 7742 Processor (64 MB cache, 2.25 GHz) with 512 GB RAM running Debian Linux 11.5.Each simulation time step takes approximately 0.4 seconds of wall-clock time when run on in parallel on all cores of a 64-core AMD EPYC 7742 processor.

### From sample to computational domain

**Fig 1:**
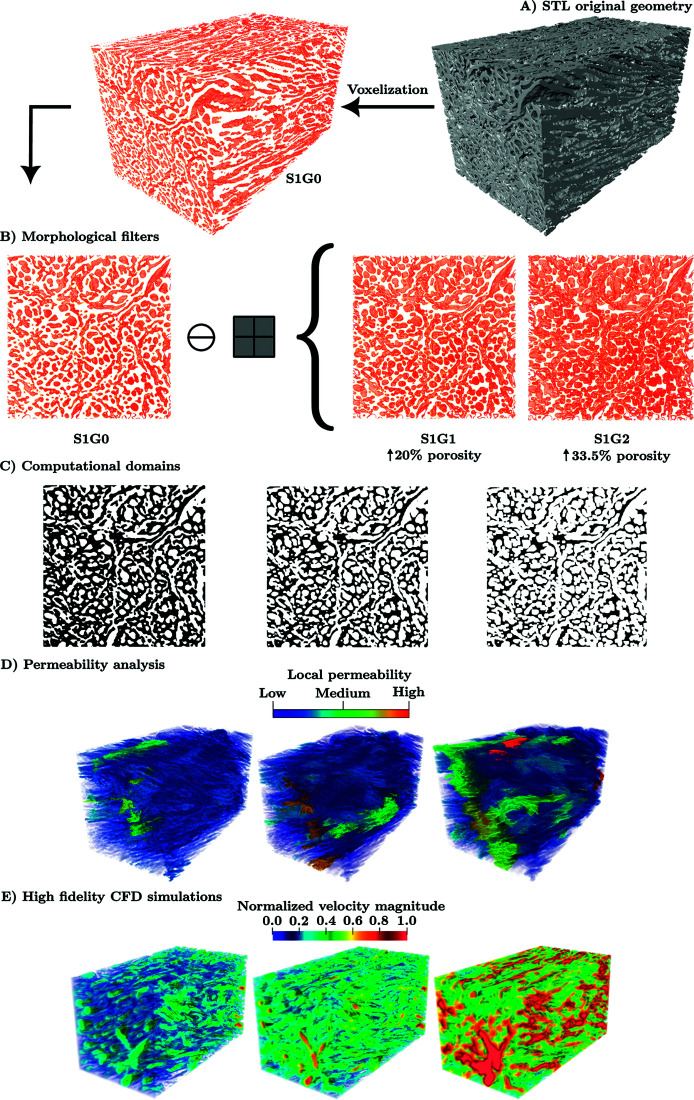
Workflow outline for investigating the effect of microstructural properties on fluid flow in human meniscus: (A) The 3D STL geometry of the microstructure volume of the human meniscus (S1G0) is constructed from high-resolution *μ*-CT (6.25*μ*m). **(B)** The meniscus channels are eroded by applying morphological filters (erosion) to obtain two additional geometries ( **S1G1** and **S1G2**) with different microstructural properties. This controlled erosion filter changes the microstructural properties of the meniscus, mainly the porosity. The resulting geometries allow us to study the effect of the meniscus’s wall degradation on its functionality, as shown in Table 1. **(C)** The computational domain of the different meniscus geometries ( **S1G0**, **S1G1**, and **S1G2**) with implicit boundary representation is created using an in-the Brinkman penalisation. ( **D**) Flux analysis at the pore scale for the three structures yields a distribution of local permeability. The local permeability is characterised by the volume of voxel clusters of the porous network with pore diameters exceeding 4 voxels. Blue regions indicate areas of lower local permeability and correspond to smaller volume magnitudes. Conversely, red regions represent higher local permeability, signifying areas with greater volume magnitudes. ( **E)** High-fidelity computational fluid dynamics (CFD) flow simulations are conducted using the Discretisation-Corrected Particle Strength Exchange (DC-PSE) method. All flow velocities in the three panels are all normalised to the maximum velocity magnitude observed in S1G0. Evolution of the velocity field with respect to the porosity: from **S1G0** to **S1G1** and **S1G2**, the porosity increases by 20.5% and 33%, respectively, and the maximal velocity increases by 30% and 120%, respectively.

As previously mentioned, the reference samples in the present study were obtained from human meniscus donors. A brief description of this procurement is presented to illustrate the transition from the in vivo state to preservation and observation. In the study by Agustoni et al. [10], two meniscus samples were extracted, here identified as **S1G0** and **S2G0**, our two reference samples. To obtain better image contrast, the samples were lyophilized prior to the *μ* -CT (6.25, *μ*m resolution) scans and therefore contained only two phases: solid and void. It has been previously shown [10] that the specific freeze-drying procedure preserves the range of pore sizes compared to the results obtained by Vetri et al. [36] using confocal multiphoton microscopy. We cannot exclude the existence of an artificial porosity induced by this experimental procedure, as we acknowledge that no observation technique is without limitations. The *μ* CT scans were then used to generate the 3D volume in the form of the Standard Triangle Language (STL) format geometry of the meniscus specimens used as input for our work, Fig 1A. The ex vivo sample **S1G0** had a diameter of 3.5mm and a length of 4.6mm. The generated STL had 5.68 million vertices and 11.55 million faces. The ex vivo sample **S2G0** had a diameter of 1mm and a length of 3.13mm. The resulting STL geometry had 3.55 million vertices and 7.14 million faces (see From Sample to Computational Domain). The different sizes of the two samples, with **S1G0** having a diameter 3.5 times larger than that of **S2G0**, are due to the natural dimensions of the tissues extracted from the donors.

STL geometries have surfaces that represent the walls of the meniscus, but these surfaces cannot be used directly in most numerical methods. To overcome this, we use an algorithm that labels particles with a mask field χ(x) [37,38] (or characteristic function) differentiating fluid or solid phase depending on their position, x, within the STL surface. To study the mechanical behavior of the meniscus with varying porosity, the original geometries **S1G0** and **S2G0** are taken as a reference, and modified geometries are generated with different volume fractions while conserving the connectivity of the porous network through a homogeneous erosion process. This process involves STL-to-voxel conversion, morphological erosion of the stack of voxels, voxel-to-STL conversion, and decimation and formatting to reduce computational cost.

## Results

In Fig 1 we outline the main steps of this work. From the previous content in the Introduction it is evident that there is a clinical need to study the effect of the meniscus wall degradation on its functionality, thus, demonstrating the pertinence of the present work. Meniscus degeneration, however, is a multi-faceted process that encompasses not just geometric alterations to the microstructure, but also changes in the biochemical composition, material properties, and structural organisation of the meniscus. Understanding meniscus degeneration will therefore ultimately require a holistic approach that goes beyond morphological alterations [39,40]. As a first step, we here focus on the effect of microstructure geometry on fluid mechanics in the meniscus, which our computational model enables us to study in isolation. For this, we apply a uniform erosion morphological filter with two different characteristic lengths to **S1G0**, modifying its porosity to obtain two new “degraded” meniscus geometries ( **S1G1** and **S1G2**) while preserving the connectivity of the porous network, Fig 1(B). The 3D volume of the three geometries is then represented in the mesh-free computational domain as particles (point clouds). Due to the complexity of the meniscus geometry, the Brinkman penalisation technique is applied for implicit representation of the complex boundaries [37], Fig 1(C). In Fig 1(D) the local permeability distribution of the three porous geometries is presented. This distribution allows the identification of areas in which the flow will tend to move more easily (high local permeability), see Fig 1(D) and inline with our numerical results in Fig 1(E), further validating the significance of local permeability in influencing fluid flow behavior within the porous medium. The Entropically Damped Artificial Compressibility (EDAC) solver for The Discretisation-Corrected Particle Strength Exchange (DC-PSE) [31] is utilised to model the three dimensional viscous fluid flow inside the meniscus by solving the EDAC formulation, in Fig 1(E). Finally, we examine the the impact of inlet pressure and dynamic viscosity on fluid flow, considering various porosity values across different microstructures, Figs 4 and 5.

**Fig 2:**
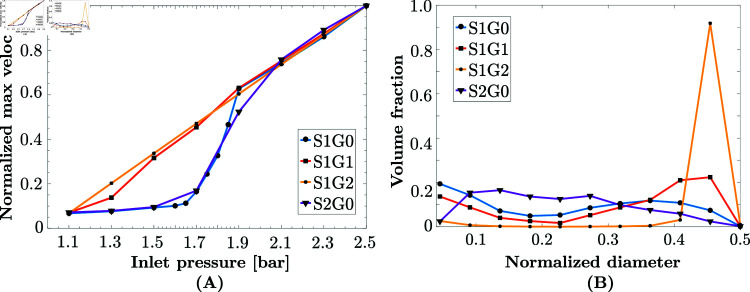
(A) The Normalised maximum flow velocity vs. inlet pressure; original geometry S1G0 (•, blue); eroded geometry S1G1 (∎, red); eroded geometry S1G2 (×, yellow); original geometries 2 S2G0 (▾, purple). **S1G0** and **S2G0** show a threshold behavior with respect to the inlet pressure, in accordance with the flow regimes described by Fithian et al. [6]. The eroded geometries **S1G1** and **S1G2** lose this property, but keeping the same behavior at high pressures. **(B)** The volume fraction occupied by different normalised pore diameters. The wide distribution of pore diameters in **S1G0** (•, blue) and **S2G0** (▾, purple) indicates a high level of heterogeneity, while the peak at 0.9 in **S1G2** (×, yellow) indicates a relatively homogeneous geometry.

**Fig 3:**
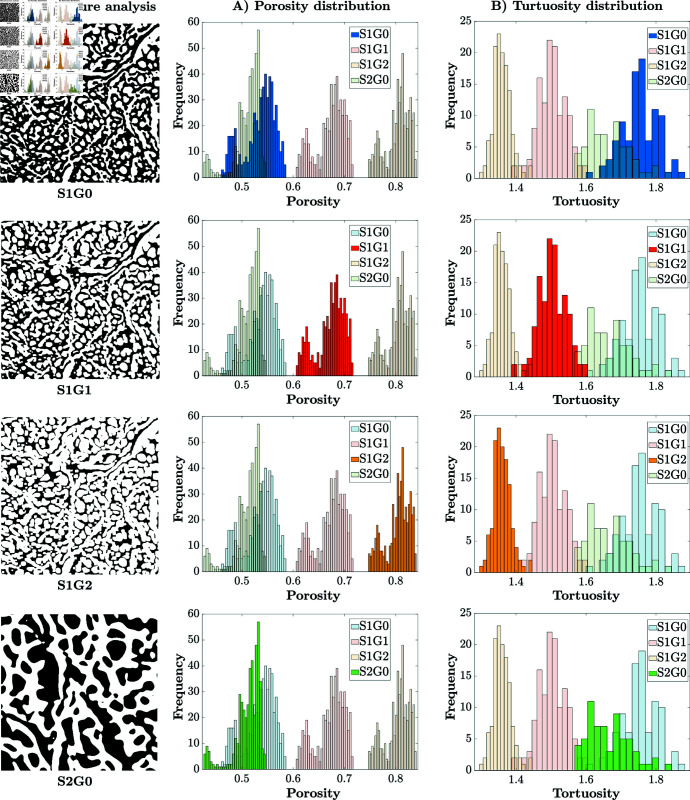
Comparative analysis of four distinct microstructures: S1G0, S1G1, S1G2, and S2G0. Each row show the microstructure, followed by its porosity and tortuosity. Histograms for the respective sample are in full color, whereas those of the other samples shown transparently for comparison. The porosity of **S1G0** and **S2G0** (0.53%±0.05%) aligns with *μ*CT measurements from the literature. Note the increase in porosity in **S1G1** and **S1G2** due to the morphological filter. The mean value of tortuosity for **S1G0** and **S2G0** is close to 2, a typical value for living tissues [41–43]. As the porosity increases in **S1G1** and **S1G2**, the tortuosity decreases, indicating a more permeable porous medium.

**Fig 4:**
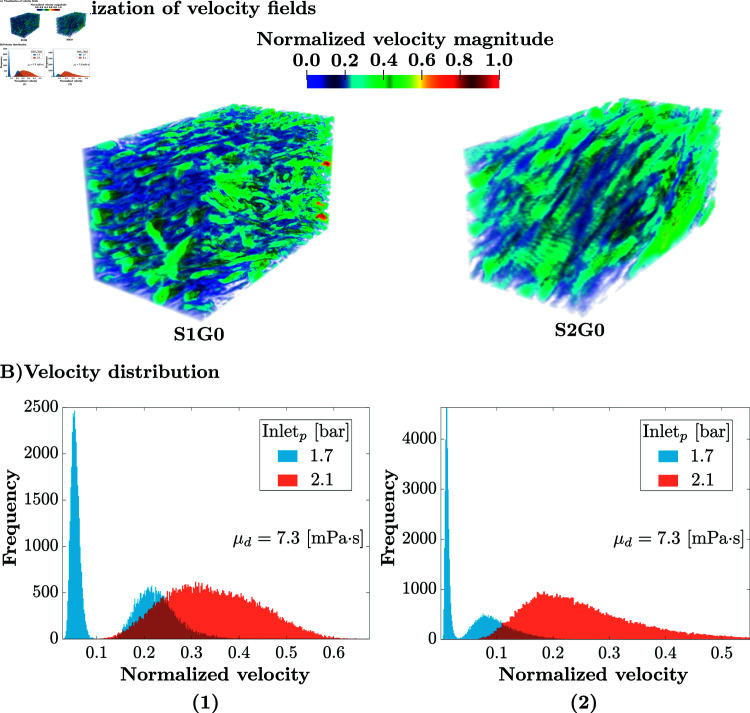
(A) Visualisation of the normalised (to the maximum value in S1G0) flow velocity fields for S1G0 and S2G0, with inlet pressure 2.1bar. **(B)** flow velocity distribution histograms; in blue, inlet pressure 1.7bar; in red, 2.1bar. The velocity is normalised with the maximum velocity observed for inlet pressure $P_{\text{inlet}}=2.1\;\text{bar}$, with the reference viscosity $\mu =7.3\cdot 10^{-3}\;\text{Pa}\cdot \text{s}$. **(B-1)** The histogram of the non-zero velocity field for **S1G0**. The flow presents a general trend toward extremely small velocities (visco-elastic), more than 50% of the porosity contains flow below 5% of maximal velocity ($1.53\;\cdot \;10^{-3}\;\text{m}/\text{s}$). The synovial fluid transitions to porous-permeable behavior, which means that the flow is dominated by the pressure gradient, at super-critical pressure. The velocity distribution then becomes more Gaussian in shape and centered at around 33% of the maximal velocity. **(B-2)**The same histograms for sample **S2G0**, showing the threshold behavior even more clearly.

**Fig 5:**
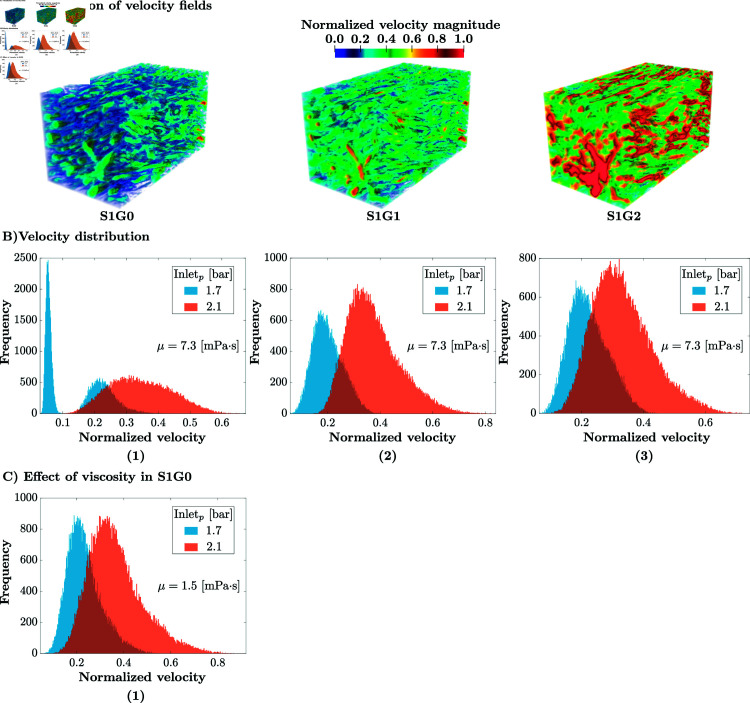
(A) Visualisation of the normalised (to the maximum value in S1G0) flow velocity fields for S1G0, S1G1 and S1G2, with inlet pressure 2.1bar. The normalised velocity in **S1G2** is 120% higher than in **S1G0**, whereas it is only 30% higher in **S1G1**, highlighting the nonlinear effect of pore size on flow dynamics in the meniscus. **(B)** flow velocity distribution histograms; in blue, inlet pressure 1.7bar; in red, 2.1bar. The velocity is normalised with the maximum velocity observed for inlet pressure $P_{\text{inlet}}=2.1\;\text{bar}$, with the reference viscosity $\mu =7.3\;\cdot \;10^{-3}\;\text{Pa}\;\cdot \;\text{s}$. **(B-1)** The flow velocity distribution histogram of the non-zero velocity field for **S1G0**. The flow presents a general trend toward extremely small velocities (visco-elastic), more than 50% of the porosity contains flow below 5% of maximal velocity ($1.53\;\cdot \;10^{-3}\;\text{m}/\text{s}$). The synovial fluid transitions to porous-permeable behavior, which means that the flow is dominated by the pressure gradient, at super-critical pressure. The velocity distribution then becomes more Gaussian in shape and centered at around 33% of the maximal velocity. **(B-2)** The histogram of the non-zero velocity field for sample **S1G1**. **(B-3)** The histogram of the non-zero velocity field for sample **S1G2**. The results of the two previous structures suggest that the geometrical properties of the meniscus significantly affect the behavior of fluid under varying pressure. Here, the threshold behavior is lost in both samples with flow always in the porous-permeable regime. **(C-1)** The flow velocity histograms for **S1G0** with a lower synovial fluid viscosity $\mu =1.5\;\cdot \;10^{-3}\;\text{Pa}\;\cdot \;\text{s}$, characteristic of osteoarthritis [29], where the dual behavior is lost. This confirms that the two flow regimes observed in the healthy samples are not solely due to the geometry of the samples, but also depend on the physiological properties of the synovial fluid, specifically as its viscosity.

### Geometric parameter analysis

Before analysing three crucial geometrical parameters, namely porosity, average tortuosity, and connectivity, it is necessary to examine how the microstructural properties of the meniscus impact its functionality.

We modify the healthy sample **S1G0** outside its physiological configurations, the sample undergoes a morphological filter (erosion filter) to artificially increase the porosity by increasing the channel diameters. This filter has a significant impact on the properties of the porous medium, see Table 1. In the table, $\ell_{c}$ represents the structural element length, which corresponds to the size of the voxel being eroded. Applying the erosion filter of length (structural element $\ell_{c}$) 2 and 4 voxels, the porosity of **S1G0** is artificially increased by 20.5% and 33.5%, respectively. This leads to the creation of two new meniscus geometries, **S1G1** and **S1G2**, as presented in Fig 1(B) and 1(C). Tortuosity provides a measure of the difficulty level for fluid to flow within a porous network. When the length scale of the erosion filter is increased, the average tortuosity decreases, indicating that the porous network becomes more permeable.

The non-connected clusters in a porous medium refer to isolated regions within the sample where there is no possibility of fluid flow due to a lack of connectivity to other regions of the sample. In other words, these clusters represent areas where fluid flow is hindered or impossible. The numbers provided in Table 1, in the volume fraction $v_{f}$ of connected voids column, indicate that the porous network in the original geometries **S1G0** and **S2G0** have a high degree of connectivity, >99.7% of the volume fraction. This is important for a living tissue, as it ensures that there are no “dead” regions without fluid exchange. This high degree of connectivity is preserved also in the eroded geometries, showing that the sample porosity can be modified without significantly altering the overall connectivity of the porous network (note that for **S2G0**, the number of non-connected clusters is representative of a smaller volume compared to **S1G0**), see Table 1.

**Table 1:** Computed volume fraction $v_{f}$ and microstructure statistics for the simulation domains. It is important to highlight the significant size difference between the two samples, with S1G0 having a diameter that is 3.5 times larger than that of S2G0.

Geometry	Erosion length of the structural element $\ell_{c}$	Average porosity	Non-connected clusters	$v_{f}$ of connected voids	Average tortuosity
S1G0	0	53.45%	247	0.997377	2.4
S1G1	2	67.23%	180	0.999631	1.7
S1G2	4	80.36%	69	0.999928	1.4
S2G0	0	51.08%	92	0.998975	2.2

The volume fraction in relation to the normalised pore diameters is shown in Fig 2(B), which provides insight into the homogeneity or heterogeneity of the pore structure. The wide distribution of pore diameters in **S1G0** and **S2G0** indicates a high level of heterogeneity, while the peak at 0.9 of the maximum diameter in **S1G2** indicates a high level of homogeneity.

The histograms and fitted probability density functions for porosity, along with the average tortuosity, are presented for the four geometries in Fig 3. The histograms provide insights into the distribution of porosity and tortuosity within each microstructure, revealing unique characteristics and variations among the studied microstructures.

We note that the porosity of the original geometry (53±5%), obtained by *μ*CT scans analysis, is within the range of the current literature (from 34.1% [41] to 65% [42]). We also note that the mean value of tortuosity in the original geometries is 2.3, close to the value of 2, commonly used in the porous medium literature for living tissues [43–45]. As the porosity increases in **S1G1** and **S1G2** the tortuosity decreases, indicating a more permeable porous medium.

### The effect of inlet pressure on the flow regime

The focus of our study is to investigate how fluid flow is affected by varying pressure at the inlet, with different microstructural properties (healthy and pathological menisci). To accomplish this, we apply initial pressure boundary conditions at the inflow using a range of values from 1.1 to 2.5bar (see Simulation setup, initial condition and boundaries). According to Fithian et al. [6], synovial fluid can exhibit two types of flow behavior: (1) visco-elastic behavior, which is mediated by proteoglycans of the extra-cellular matrix, (2) porous-permeable behavior, which occurs when the synovial fluid is subjected to flow through a hydraulic pressure gradient or matrix compaction.

The results of our numerical simulations are summarised in Fig 2(A), where those two regimes of the velocity with respect to inlet pressure are clearly seen for the original geometries **S1G0** and **S2G0**. It is clear that only the original, healthy geometries **S1G0** and **S2G0** exhibit two flow regimes with a threshold of around 1.7bar inlet pressure. This threshold behavior is not seen in any of the eroded geometries **S1G1** and **S1G2**, where porosity no longer limits fluid flow.

In healthy samples ( **S1G0** and **S2G0)** this surprising qualitative change in behavior is not an artifact of the mathematical flow model used (see Mathematical modelling), as the pressure-velocity relationship is locally linear, but an intrinsic geometrical property of the original sample itself and the physiological viscosity of the synovial fluid. For a better understanding of this behavior, the histograms of velocities with an inlet pressure of 1.7 and 2.1 bar for **S1G0** are provided in Fig 4(B-1). The simulated flow for sub-critical inlet pressure (blue in Fig 4), presents a general trend toward extremely small velocities (visco-elastic). Specifically, in **S1G0**, more than 50% of the porosity contains flow below 5% of maximal velocity ($1.53\;\cdot \;10^{-3}\;\text{m}/\text{s}$), and this trend is even more obvious for **S2G0** (Fig 4(B-2)). Above the threshold of 1.7 bar, (red in Fig 4), the model predicts a porous-permeable flow behavior, which means that the flow is dominated by the pressure gradient. The velocity distribution becomes more Gaussian in shape and centered around about 33% of the maximal velocity. This is a clear indication that the flow behavior has transitioned to the porous-permeable regime.

The synovial dynamic viscosity has an impact on the mechanical function of the meniscus. A viscosity below the physiological range is for example observed, for example, in cases of osteoarthritis [29]. This change in viscosity affects the flow behavior of the synovial fluid, as evidenced by the loss of the threshold behavior observed under healthy conditions, see Fig 5(C-1). This confirms that the dual flow behavior is not solely influenced by the geometry of the samples, but also by the physiological properties of the synovial fluid.

The flow transition also disappears upon meniscus degeneration, modeled by an increased porosity (+20.5% for **S1G1**, accompanied by a -29.1% decrease in tortuosity), see Fig 5(B-2). The meniscus then shows a linear flow behavior with respect to the inlet pressure. The same is observed in **S1G2** (porosity increases of +33.5% and tortuosity decreases of -41.6%), see Fig 5(B-3). Since no other simulation parameter changed, this qualitative change can be attributed purely to intrinsic geometrical properties of the samples at physiological values of synovial fluid viscosity. These results are summarised Fig 2(A), where the threshold behavior of velocity with respect to inlet pressure is only visible for the original healthy geometries ( **S1G0** and **S2G0**).

### The effect of the pore size on the maximum flow velocity

We perform numerical simulations of the fluid dynamics of the synovial fluid inside the fully-resolved porous microstructure geometries of two human meniscus samples **S1G0** and **S2G0**. The details of the simulation method are given in the Materials and Methods section and have been verified and validated elsewhere [31].

In order to see how the average pore diameter influences the fluid flow velocity field, we also consider the two eroded versions of sample **S1G0**, namely **S1G1** and **S1G2** (cf. Table 1). Visualisations of the simulation results for an inlet pressure of 2.1bar are shown in Fig 5(A). They qualitatively agree with the experimental findings of Proctor et al. [8], who provoked porous-permeable flows in meniscus samples through consolidation experiments at 2bar. All cases in Fig 5(A) have the velocity magnitudes normalised by the same maximum velocity obtained in **S1G0**. The effect of pore size on fluid flow is clearly visible.

In **S1G2**, the normalised velocity prediction is 120% higher than in **S1G0**, whereas in **S1G1**, the normalised velocity is only 30% higher than in **S1G0**. Using the erosion filter, the porosity increased by 20.5% in **S1G1** and 33.5% **S1G2**. Therefore, it is evident that the porosity of the meniscus medium has a significant and non-linear impact on fluid flow. This finding emphasises the importance of understanding the relationship between porosity and velocity in fluid flow.

The nonlinear flow characteristics can influence how effectively the meniscus distributes loads and lubricates the knee joint. Understanding these nonlinearities can provide insights into optimal joint function and highlight potential issues in case degeneration.

## Discussion

To advance our knowledge of the biomechanics of the knee joint, it is critical to investigate the microstructure interactions of the human meniscus and how it affects its mechanical function reflected on its macroscopical response. Connecting the microstructural properties of the meniscus to its macroscopic function is still an unsolved problem, and investigating the impact of porosity and connectivity of the porous network within the meniscus on macroscopic fluid-mechanical properties can provide insights into physiological mechanisms. We therefore reconstructed the microstructure of two human menisci using high-resolution *μ*-CT scans (6,25*μ*m). By computational fluid dynamics (CFD) flow simulations with mesh-free particle methods and implicit boundaries, we show that the absorption properties of the meniscus are mainly explained by the diameters of the channels. For instance, a 30% increase in porosity leads to a 120% increase in velocity magnitude. These findings support the experimentally observed fact that the degraded function of aged menisci is associated with a condensed collagen network. Our findings may have implications for the treatment of knee injuries and aging, and contribute to a better understanding of meniscal biomechanics.

We used two *μ*CT scans (6.25μm resolution) of human meniscus samples [10] to study the effect of microstructure on meniscus fluid dynamics. We developed an end-to-end computational pipeline from *μ*CT scans to computational simulation results. This was then used to simulate pore-scale flow using a mesh-free simulation method with different inlet pressure conditions.

For healthy sample geometries under physiological conditions, our simulations were able to reproduce two regimes of flow in accordance with the biophysical literature [6,8,11]: a visco-elastic creeping flow below a critical pressure of about 1.7bar, and laminar porous-permeable flow above the threshold pressure. Interestingly, this threshold behavior was lost in both degraded sample geometries and at lower synovial fluid dynamic viscosity, both symptomatic of diseased states of the meniscus.

Fully resolved computer simulations of healthy menisci allowed us to understand the effect of the inlet pressure on flow patterns. This validated the method by comparison with experimental findings [6,8,11]. We then modified the healthy sample geometries outside the physiological range to study the effect of degeneration of the meniscus wall, specifically of a decrease of collagen content and increase of water content [28].

This was achieved by using an erosion filter to simulate porosity volume fractions ranging from 53% to 80% at constant network connectivity. For the absolute porosity, we found average values between 100 *μ*m for **S1G0** and 166 *μ*m for **S1G2** with corresponding modes between 5.56 *μ*m and 27.82 *μ*m. All distributions had a shifted half-Gaussian-like shape. The distance between mode and mean values suggests that in different sections of the effective direction of the flux, multiple smaller porosity channels are intersected.

The resulting artificially eroded meniscus geometries were comparable to those seen from collagen fibers condensing in aging menisci or osteoathritis [23,28,46]. The prescribed load of the body remaining the same, this increase in permeability will shorten the consolidation time of the porous medium, leading to a joint that is more susceptible to damage. The simulation results showed that degenerating the meniscus wall non-linearly increases the transport velocity (+120% when increasing porosity by +33%) and decreases the meniscus’ tortuosity. The former impairs the meniscus’ functionality as a shock absorber, whereas the latter could hamper biochemical exchange in the tissue. According to our co-author Prof. MD. Seil, an expert in knee surgery, the erosion process identified in our research offers a potential translation for the reduction in cartilage and menisci thickness observed in MRI studies of individuals who participate in long-distance running [47]. This phenomenon is often associated with repetitive impact loading, especially in runners with intact menisci.

Our results showed that the pore size is critical in determining the fluid flow behavior, as smaller pores lead to capillary action slowing the flow, leading to a more uniform velocity distribution across pore sizes. Larger pores, on the other hand, reduce the fluid-solid interfacial area, resulting in reduced frictional forces and viscous drag, which leads to an increase in fluid velocity [48,49]. However, the exact behavior can be influenced by the nature of the fluid (e.g., synovial fluid’s viscosity) and the specific microstructure of the porous media.

We were also able to simulate the effect of osteoarthritis , which provokes a decrease in synovial fluid viscosity [29]. Indeed, our numerical results show that, if the dynamic viscosity of the synovial fluid is below physiological range, the threshold behavior of the interstitial flow, *i.e.* visco-elastic *vs.* porous-permeable, is lost. This finding is confirmed by studies, which showed that infiltration of intra-articular hyaluronate with rheological properties close to those of healthy synovial fluid helps restore the synovial fluid properties in patients with osteoarthritis [29]. Therefore, this dual behavior of the meniscus seems to be contained by the porous medium as a whole: the geometry of the microstructure coupled with the physiological range of synovial fluid dynamic viscosity.

Several leads for future improvement may be considered. First, experimental techniques have limitations we can not exclude the existence of artificial porosity provoked by freeze-drying protocol. However, the presented samples **S1G0** and **S2G0** show porosities within the physiological range of the knee meniscus [42], please refer to Table 1. The present study reproduced the biophysical behavior of the human meniscus on only two samples. Reproduction of these results on a larger cohort is critical. Second, meniscus is considered in this study as a porous system composed of a rigid solid scaffold (i.e. a network of collagen fibers is not deformed) perfused by a viscous fluid. The present methodology could be used to confirm the hypothesis by Fithian et al. [6] that the deformation of the extra-cellular matrix may provoke porous-permeable flow. In order to reproduce this phenomenon, our mathematical model would need to be extended to consider poro-elastic effects [50], which are likely to play a key role there. In the present work, we also assumed that the pores are empty due to prior freeze-drying of the samples. We also did not model details of the solid scaffold of the meniscal tissue, such as the collagen network. Our model therefore did not include additional friction forces, but studied the role of microstructure geometry in isolation. Finally, the present simulations only considered static meniscus geometries. They did therefore not allow us to study pore collapse or recovery of the tissue from load, for which a simulation in a deforming geometry would be required. Future work could extend the algorithm in this direction, albeit this is not trivial.

To conclude, analysing fluid flow behavior in different realistic meniscus geometries using a direct numerical simulation method on a parallel multi-core computer allowed us to gain insight into how changes in porosity, connectivity, pore diameter, and fluid viscosity affect the macroscopic functional properties of human menisci. Our numerical simulations indicate that the observed threshold behavior of the meniscus as a porous medium is the result of a precise balance provided by the geometry of the microstructure and the dynamic viscosity of the synovial fluid. In the future, this knowledge and the presented numerical simulation program can be used to design better prosthetics and rehabilitation protocols, offering more effective and personalised treatment options.

## Supporting information

**S1G0 Data. Meniscus sample S1G0.** The hdf5 file representing the original sample S1G0.

**S2G0 Data. Meniscus sample S2G0.** The hdf5 file representing the original sample S2G0.

**S1G1 Data. Meniscus sample S1G1.** The hdf5 file representing the eroded meniscus sample S1G1.

**S1G2 Data. Meniscus sample S1G2.** The hdf5 file representing the eroded meniscus sample S1G2.

**Code. The main code.** The code file to repreduce the results.

## References

[1] Seedhom BB, Hargreaves DJ. Transmission of the load in the knee joint with special reference to the role of the menisci: Part II: Experimental results, discussion and conclusions. Eng Med. 1979;8(4):220–8.

[2] Kurosawa H, Fukubayashi T, Nakajima H. Load-bearing mode of the knee joint: physical behavior of the knee joint with or without menisci. Clin Orthop Relat Res. 1980;(149):283–90. 7408313

[3] Burr DB, Radin EL. Meniscal function and the importance of meniscal regeneration in preventing late medical compartment osteoarthrosis. Clin Orthop Relat Res. 1982;(171):121–6. 6897209

[4] Andrews S, Shrive N, Ronsky J. The shocking truth about meniscus. J Biomech. 2011;44(16):2737–40. doi: 10.1016/j.jbiomech.2011.08.026 21924725

[5] Fox AJS, Bedi A, Rodeo SA. The basic science of human knee menisci: structure, composition, and function. Sports Health. 2012;4(4):340–51. doi: 10.1177/1941738111429419 23016106 PMC3435920

[6] Fithian DC, Kelly MA, Mow VC. Material properties and structure-function relationships in the menisci. Clin Orthop Relat Res. 1990;(252):19–31. 2406069

[7] Voloshin AS, Wosk J. Shock absorption of meniscectomized and painful knees: a comparative in vivo study. J Biomed Eng. 1983;5(2):157–61. doi: 10.1016/0141-5425(83)90036-5 6687914

[8] Proctor CS, Schmidt MB, Whipple RR, Kelly MA, Mow VC. Material properties of the normal medial bovine meniscus. J Orthop Res. 1989;7(6):771–82. doi: 10.1002/jor.1100070602 2677284

[9] Mansour JM, Mow VC. The permeability of articular cartilage under compressive strain and at high pressures. J Bone Joint Surg Am. 1976;58(4):509–16. 1270471

[10] Agustoni G, Maritz J, Kennedy J, Bonomo FP, Bordas SPA, Barrera O. High resolution micro-computed tomography reveals a network of collagen channels in the body region of the knee meniscus. Ann Biomed Eng. 2021;49(9):2273–81. doi: 10.1007/s10439-021-02763-6 33829363 PMC8455383

[11] Galandáková A, Ulrichová J, Langová K, Hanáková A, Vrbka M, Hartl M, et al. Characteristics of synovial fluid required for optimization of lubrication fluid for biotribological experiments. J Biomed Mater Res B Appl Biomater. 2017;105(6):1422–31. doi: 10.1002/jbm.b.33663 27086677

[12] Makris EA, Responte DJ, Paschos NK, Hu JC, Athanasiou KA. Developing functional musculoskeletal tissues through hypoxia and lysyl oxidase-induced collagen cross-linking. Proc Natl Acad Sci U S A. 2014;111(45):E4832-41. doi: 10.1073/pnas.1414271111 25349395 PMC4234579

[13] Gu KB, Li LP. A human knee joint model considering fluid pressure and fiber orientation in cartilages and menisci. Med Eng Phys. 2011;33(4):497–503. doi: 10.1016/j.medengphy.2010.12.001 21208821

[14] Kazemi M, Li LP. A viscoelastic poromechanical model of the knee joint in large compression. Med Eng Phys. 2014;36(8):998–1006. doi: 10.1016/j.medengphy.2014.04.004 24933338

[15] Seyfi B, Fatouraee N, Imeni M. Mechanical modeling and characterization of meniscus tissue using flat punch indentation and inverse finite element method. J Mech Behav Biomed Mater. 2018;77:337–46. doi: 10.1016/j.jmbbm.2017.09.023 28965040

[16] Maritz J, Agustoni G, Dragnevski K, Bordas SPA, Barrera O. The functionally grading elastic and viscoelastic properties of the body region of the knee meniscus. Ann Biomed Eng. 2021;49(9):2421–9. doi: 10.1007/s10439-021-02792-1 34075449 PMC8455388

[17] Iiyama T, Nishikawa K, Suzuki TM, Kaneko K. Study of the structure of a water molecular assembly in a hydrophobic nanospace at low temperature with in situ X-ray diffraction. Chem Phys Lett. 1997;274:152–8.

[18] Wildenschild D, Sheppard AP. X-ray imaging and analysis techniques for quantifying pore-scale structure and processes in subsurface porous medium systems. Adv Water Res. 2013;51:217–46.

[19] Seymour JD, Gage JP, Codd SL, Gerlach R. Magnetic resonance microscopy of biofouling induced scale dependent transport in porous media. Adv Water Res. 2007;30(6):1408–20.

[20] Nair M, Shepherd JH, Best SM, Cameron RE. MicroCT analysis of connectivity in porous structures: optimizing data acquisition and analytical methods in the context of tissue engineering. J R Soc Interface. 2020;17(165):20190833. doi: 10.1098/rsif.2019.0833 32316883 PMC7211477

[21] Vidotto M, Bernardini A, Trovatelli M, De Momi E, Dini D. On the microstructural origin of brain white matter hydraulic permeability. Proc Natl Acad Sci U S A. 2021;118(36):e2105328118. doi: 10.1073/pnas.2105328118 34480003 PMC8433514

[22] Hasan S, Niasar V, Karadimitriou NK, Godinho JRA, Vo NT, An S, et al. Direct characterization of solute transport in unsaturated porous media using fast X-ray synchrotron microtomography. Proc Natl Acad Sci U S A. 2020;117(38):23443–9. doi: 10.1073/pnas.2011716117 32900944 PMC7519338

[23] Tsujii A, Nakamura N, Horibe S. Age-related changes in the knee meniscus. Knee. 2017;24(6):1262–70. doi: 10.1016/j.knee.2017.08.001 28970119

[24] Vikingsson L, Claessens B, Gómez-Tejedor JA, Gallego Ferrer G, Gómez Ribelles JL. Relationship between micro-porosity, water permeability and mechanical behavior in scaffolds for cartilage engineering. J Mech Behav Biomedical Materials. 2015;48:60–9. 10.1016/j.jmbbm.2015.03.021 25913609

[25] Wang X, Ding Y, Li H, Mo X, Wu J. Advances in electrospun scaffolds for meniscus tissue engineering and regeneration. J Biomed Mater Res B Appl Biomater. 2022;110(4):923–49. doi: 10.1002/jbm.b.34952 34619021

[26] Tarafder S, Park G, Lee CH. Explant models for meniscus metabolism, injury, repair, and healing. Connect Tissue Res. 2020;61(3–4):292–303. doi: 10.1080/03008207.2019.1702031 31842590 PMC7190414

[27] Rai MF, Brophy RH, Rosen V. Molecular biology of meniscus pathology: lessons learned from translational studies and mouse models. J Orthop Res. 2020;38(9):1895–904. doi: 10.1002/jor.24630 32068295 PMC7802285

[28] Kestilä I, Folkesson E, Finnilä MA, Turkiewicz A, Önnerfjord P, Hughes V, et al. Three-dimensional microstructure of human meniscus posterior horn in health and osteoarthritis. Osteoarthritis Cartilage. 2019;27(12):1790–9. doi: 10.1016/j.joca.2019.07.003 31301431 PMC7610688

[29] Nicholls M, Manjoo A, Shaw P, Niazi F, Rosen J. A comparison between rheological properties of intra-articular hyaluronic acid preparations and reported human synovial fluid. Adv Ther. 2018;35(4):523–30. doi: 10.1007/s12325-018-0688-y 29542009 PMC5910459

[30] Clausen JR. Entropically damped form of artificial compressibility for explicit simulation of incompressible flow. Phys Rev E Stat Nonlin Soft Matter Phys. 2013;87(1):013309. doi: 10.1103/PhysRevE.87.013309 23410462

[31] Singh A, Sbalzarini IF, Obeidat A. Entropically damped artificial compressibility for the discretization corrected particle strength exchange method in incompressible fluid mechanics. Comput Fluids. 2023;267:106074.

[32] Schrader B, Reboux S, Sbalzarini IF. Discretization correction of general integral PSE operators for particle methods. J Comput Phys. 2010;229:4159–82.

[33] Fu J, Ni M, Chai W, Li X, Hao L, Chen J. Synovial fluid viscosity test is promising for the diagnosis of periprosthetic joint infection. J Arthr. 2019;34(6):1197–200. 10.1016/j.arth.2019.02.009 30837099

[34] Bera K, Kiepas A, Godet I, Li Y, Mehta P, Ifemembi B, et al. Extracellular fluid viscosity enhances cell migration and cancer dissemination. Nature. 2022;611(7935):365–73. doi: 10.1038/s41586-022-05394-6 36323783 PMC9646524

[35] Incardona P, Leo A, Zaluzhnyi Y, Ramaswamy R, Sbalzarini IF. OpenFPM: a scalable open framework for particle and particle-mesh codes on parallel computers. Comp Phys Commun. 2019;241:155–77.

[36] Vetri V, Dragnevski K, Tkaczyk M, Zingales M, Marchiori G, Lopomo N, et al. Advanced microscopy analysis of the micro-nanoscale architecture of human menisci. Scientific Reports. 9. 10.1038/s41598-019-55243-2 PMC6904744 31822796

[37] Obeidat A, Bordas SPA. An implicit boundary approach for viscous compressible high Reynolds flows using a hybrid remeshed particle hydrodynamics method. J Comput Phys. 2019;391:347–64.

[38] Obeidat A, Andreas T, Bordas SPA, Ziliana A. Discrete filters for viscous compressible high Reynolds flows in industrial complex geometry using a hybrid remeshed particle hydrodynamics method. in print.

[39] Otani S, Kanamoto T, Oyama S, Yamakawa S, Shi W, Miyazaki R. Meniscus surface texture is associated with degenerative changes in biological and biomechanical properties. Sci Rep. 2022;12(1):2045–322. 35831482 10.1038/s41598-022-16206-2 PMC9279509

[40] Bradley PX, Thomas KN, Kratzer AL, Robinson AC, Wittstein JR, DeFrate LE. The interplay of biomechanical and biological changes following meniscus injury. Curr Rheumatol Rep. 2023;25(2):35–46. 36479669 10.1007/s11926-022-01093-3 PMC10267895

[41] Zhu S, Tong G, Xiang J-P, Qiu S, Yao Z, Zhou X, et al. Microstructure analysis and reconstruction of a meniscus. Orthop Surg. 2021;13(1):306–13. doi: 10.1111/os.12899 33403835 PMC7862168

[42] Pereira H, Caridade S, Frias A, Silva-Correia J, Pereira D, Cengiz I. Biomechanical and cellular segmental characterization of human meniscus: building the basis for tissue engineering therapies. Osteoarth Cartilage. 2014;22(9):1271–81. 10.1016/j.joca.2014.07.001 25038489

[43] Sciumè G, Santagiuliana R, Ferrari M, Decuzzi P, Schrefler BA. A tumor growth model with deformable ECM. Phys Biol. 2014;11(6):065004. doi: 10.1088/1478-3975/11/6/065004 25427284 PMC4632987

[44] Santagiuliana R, Milosevic M, Milicevic B, Sciumè G, Simic V, Ziemys A, et al. Coupling tumor growth and bio distribution models. Biomed Microdevices. 2019;21(2):33. doi: 10.1007/s10544-019-0368-y 30906958 PMC6686908

[45] Urcun S, Rohan P-Y, Skalli W, Nassoy P, Bordas SPA, Sciumè G. Digital twinning of cellular capsule technology: emerging outcomes from the perspective of porous media mechanics. PLoS One. 2021;16(7):e0254512. doi: 10.1371/journal.pone.0254512 34252146 PMC8274916

[46] Gade PS, Lee K, Pfaff BN, Wang Y, Robertson AM. Degradation and erosion mechanisms of bioresorbable porous acellular vascular grafts: an in vitro investigation. J R Soc Interface. 2017;14(132):20170102. doi: 10.1098/rsif.2017.0102 28701504 PMC5550965

[47] Mastrokalos DS, Papagelopoulos PJ, Mavrogenis AF, Hantes ME, Paessler HH. Changes of the posterior meniscal horn height during loading: an in vivo magnetic resonance imaging study. Orthopedics. 2008;31(1). 10.3928/01477447-20080101-28 19292170

[48] Armatas GS. Determination of the effects of the pore size distribution and pore connectivity distribution on the pore tortuosity and diffusive transport in model porous networks. Chem Eng Sci. 2006;61(14):4662–75. doi: 10.1016/j.ces.2006.02.036

[49] Jiang Z, Wu K, Couples GD, Ma J. The impact of pore size and pore connectivity on single-phase fluid flow in porous media. Advanced Eng Mat. 2010;13(3):208–15.

[50] Biot MA. General theory of three-dimensional consolidation. J Appl Phys. 1941;12(2):155–64.

